# Prevalence of Disability by Occupation Group — United States, 2016–2020

**DOI:** 10.15585/mmwr.mm7220a1

**Published:** 2023-05-19

**Authors:** Taylor M. Shockey, Kelsie Fox, Guixiang Zhao, NaTasha Hollis

**Affiliations:** ^1^Division of Field Studies and Engineering, National Institute for Occupational Safety and Health, CDC; ^2^Division of Population Health, National Center for Chronic Disease Prevention and Health Promotion, CDC; ^3^Office of Health Equity, Immediate Office of the Director, CDC.

In 2020, approximately 21.5 million employed U.S. adults aged 18–64 years had some form of disability. Although 75.8% of noninstitutionalized persons without disability aged 18–64 were employed, only 38.4% of their counterparts with disability were employed (*1*). Persons with disability have job preferences similar to persons without disability but might encounter barriers (e.g., lower average training or education levels, discrimination, or limited transportation options) that affect the types of jobs they hold (*2*,*3*). CDC analyzed 2016–2020 Behavioral Risk Factor Surveillance System (BRFSS) data from 35 states and Guam to estimate disability prevalences, by type and occupation group, among currently employed U.S. adults aged 18–64 years. The highest adjusted disability prevalences were among workers in three of the 22 major occupation groups: food preparation and serving-related (19.9%); personal care and service (19.4%); and arts, design, entertainment, sports, and media (17.7%). Occupation groups with the lowest adjusted disability prevalences were business and financial operations (11.3%), health care practitioners and technicians (11.1%), and architecture and engineering (11.0%). The distributions of persons with and without disability differ across occupations. Workplace programs that address the training, education, and workplace needs of employees with disability might improve workers’ ability to enter, thrive in, and advance in a wider range of occupations.

BRFSS is an annual, random-digit–dialed telephone survey of noninstitutionalized, U.S. civilian residents aged ≥18 years. Conducted by states and territories, BRFSS gathers data on health-related risk behaviors, chronic illnesses and conditions, and use of health-related services.* The BRFSS questionnaire comprises standard and rotating core questions asked by all states and territories, as well as optionally administered topical modules and state-added questions. Thirty-five states and Guam^†^ administered the optional industry and occupation module at least 1 year during 2016–2020. The median, combined mobile phone and landline response rate during the 2016–2020 survey years for all states, territories, and the District of Columbia ranged from 45.9% to 49.9%.^§^

To determine occupation, employed respondents were asked, “What kind of work do you do, for example, registered nurse, janitor, cashier, auto mechanic?”^¶^ Participants' responses were recorded as free text and later coded by an auto-coding system or computer-assisted human coders** to one of 22 two-digit standard occupational classification major groups promulgated by the U.S. Department of Labor Bureau of Labor Statistics.^††^ To assess disability, respondents were asked the six-item question set on hearing, vision, cognition, mobility, self-care, and independent living^§§^ in the BRFSS core questionnaire. Respondents replying “Yes” to at least one of these questions are considered to have a disability.

Among the 2016–2020 BRFSS participants who completed the industry and occupation optional module (1,053,331), 50.1% were currently employed and considered for analyses. Among respondents, those on active military duty (0.3%); those who were employed but reported “unpaid,” “retired,” or “disabled” as their occupation (0.1%); those who provided insufficient information to code occupation (6.9%); those who were missing information for occupation (7.4%); and adults ≥aged 65 years (11.5%) were excluded. The final analytic sample contained 395,141 respondents. Respondents with missing information for a specific disability type (2.2% missing for hearing, 2.4% for vision, 2.7% for cognitive, 2.7% for mobility, 2.7% for self-care, 2.9% for independent living, and 3.2% for any disability) were removed from the respective analyses. Prevalence of disability status and types were calculated for the 22 major occupation groups with and without adjustment for these sociodemographic variables: age group (18–24, 25–34, 35–44, 45–54, or 55–64 years), sex, race and ethnicity (non-Hispanic Black or African American, non-Hispanic White, Hispanic or Latino [Hispanic], or non-Hispanic other race or multiracial), and education level (less than high school diploma, high school diploma, some college, or college graduate or above). Adjusted prevalence estimates were obtained using log-linear regression analyses with a robust variance estimator while adjusting for sociodemographic variables. Analyses were conducted with SAS-callable SUDAAN (version 11.0.3; RTI International) to account for the complex survey design. This activity was reviewed by CDC and was conducted consistent with applicable federal law and CDC policy.^¶¶^

Overall, 14.8% of currently employed U.S. adults aged 18–64 years reported having a disability (Table 1). Cognitive disability (7.0%) was the most frequently reported disability type; self-care disability (1.0%) was least frequently reported. Prevalences of all disability types were elevated among workers who had <a high school education, were Hispanic, were veterans, lacked access to health care coverage, or had a household income <$25,000 per year. Prevalence of the following types of disability were highest among workers aged 18–24 years: vision (3.6%), cognitive (13.9%), and independent living (4.4%). Prevalences were slightly higher among women than among men for any disability (15.5% versus 14.1%), vision (2.8% versus 2.5%), cognitive (7.8% versus 6.3%), mobility (5.4% versus 3.9%), and independent living (2.8% versus 1.7%) disability.

**TABLE 1:** Unadjusted, weighted prevalence estimates of any disability and disability type* among currently employed^†^ U.S. adults aged 18–64 years, by selected characteristics — Behavioral Risk Factor Surveillance System, 35 states and Guam, 2016–2020

Characteristic	No. of respondents^§^	Disability type,^¶^ % (95% CI)						
		Hearing	Vision	Cognitive	Mobility	Self-care	Independent living	Any
**All currently employed**	**395,141**	**2.9 (2.8–3.1)**	**2.6 (2.5–2.8)**	**7.0 (6.8–7.2)**	**4.6 (4.4–4.7)**	**1.0 (0.9–1.0)**	**2.2 (2.1–2.4)**	**14.8 (14.4–15.1)**
**Age group, yrs**								
18–24	27,515	1.9 (1.6–2.3)	3.6 (3.0–4.2)	13.9 (13.1–14.9)	1.9 (1.5–2.3)	0.6 (0.4–0.8)	4.4 (3.7–5.1)	19.5 (18.4–20.7)
25–34	68,325	1.7 (1.5–2.0)	2.5 (2.2–2.7)	8.8 (8.3–9.4)	2.4 (2.2–2.7)	0.7 (0.6–0.8)	2.7 (2.4–3.0)	14.0 (13.4–14.7)
35–44	82,164	2.1 (1.9–2.4)	1.8 (1.6–2.1)	6.0 (5.7–6.4)	3.4 (3.2–3.7)	0.8 (0.7–0.9)	1.8 (1.6–2.0)	11.7 (11.2–12.2)
45–54	103,278	3.2 (3.0–3.5)	3.1 (2.8–3.3)	5.1 (4.8–5.4)	5.6 (5.2–6.0)	1.1 (0.9–1.2)	1.7 (1.5–1.9)	13.9 (13.3–14.5)
55–64	113,859	5.4 (5.1–5.8)	2.8 (2.6–3.1)	4.5 (4.2–4.8)	8.7 (8.3–9.2)	1.5 (1.3–1.7)	1.6 (1.5–1.8)	17.7 (17.1–18.3)
**Sex**								
Men	200,106	3.8 (3.5–4.0)	2.5 (2.3–2.7)	6.3 (6.0–6.6)	3.9 (3.7–4.1)	1.0 (0.9–1.1)	1.7 (1.6–1.9)	14.1 (13.7–14.5)
Women	194,880	1.9 (1.8–2.1)	2.8 (2.6–3.0)	7.8 (7.5–8.2)	5.4 (5.2–5.7)	0.9 (0.8–1.0)	2.8 (2.6–3.0)	15.5 (15.1–16.0)
**Race and ethnicity**								
Black or African American, non-Hispanic	30,214	2.0 (1.7–2.4)	4.1 (3.6–4.5)	7.3 (6.8–7.8)	5.5 (5.1–6.0)	1.1 (0.9–1.4)	1.9 (1.7–2.3)	15.4 (14.7–16.2)
White, non-Hispanic	289,478	3.1 (3.0–3.3)	1.8 (1.7–2.0)	6.5 (6.2–6.7)	4.2 (4.0–4.3)	0.8 (0.8–0.9)	2.1 (2.0–2.2)	13.6 (13.2–14.0)
Hispanic or Latino	39,744	2.8 (2.4–3.1)	4.4 (3.9–4.8)	9.1 (8.5–9.7)	5.7 (5.2–6.2)	1.3 (1.1–1.6)	2.9 (2.5–3.4)	18.8 (17.9–19.7)
Other race or multiracial, non-Hispanic	30,074	2.8 (2.2–3.5)	2.2 (1.8–2.7)	5.8 (5.2–6.4)	3.5 (3.0–4.1)	0.8 (0.6–1.0)	1.8 (1.5–2.2)	12.5 (11.5–13.7)
**Education level**								
Less than high school	20,244	4.4 (3.9–4.9)	6.4 (5.7–7.2)	12.7 (11.7–13.7)	9.2 (8.5–10.0)	2.2 (1.8–2.6)	4.1 (3.6–4.6)	25.9 (24.8–27.1)
High school	94,389	3.6 (3.3–3.9)	3.4 (3.1–3.7)	9.2 (8.7–9.6)	5.2 (4.9–5.5)	1.1 (1.0–1.2)	2.8 (2.5–3.1)	18.1 (17.5–18.7)
Some college	109,255	3.2 (3.0–3.5)	2.4 (2.2–2.6)	7.5 (7.1–7.8)	4.9 (4.6–5.3)	1.0 (0.9–1.1)	2.5 (2.3–2.7)	15.9 (15.3–16.4)
College graduate	170,562	1.7 (1.5–1.8)	1.2 (1.1–1.3)	3.3 (3.1–3.5)	2.4 (2.3–2.6)	0.4 (0.4–0.5)	1.0 (0.9–1.1)	8.0 (7.6–8.3)
**Veteran status**								
Veteran	29,471	6.5 (5.8–7.2)	2.2 (1.8–2.8)	7.1 (6.5–7.8)	5.9 (5.4–6.6)	1.4 (1.2–1.8)	2.2 (1.9–2.6)	17.5 (16.5–18.5)
Nonveteran	365,344	2.7 (2.5–2.8)	2.7 (2.5–2.8)	7.0 (6.8–7.2)	4.5 (4.3–4.6)	0.9 (0.8–1.0)	2.2 (2.1–2.4)	14.6 (14.2–14.9)
**Access to health care coverage**								
Has access to health care coverage	350,384	2.7 (2.6–2.9)	2.2 (2.1–2.4)	6.2 (6.0–6.4)	4.4 (4.2–4.5)	0.9 (0.8–0.9)	2.0 (1.9–2.2)	13.5 (13.2–13.8)
Does not have access to health care coverage	43,458	4.0 (3.6–4.4)	5.1 (4.7–5.6)	11.9 (11.2–12.7)	6.0 (5.5–6.5)	1.6 (1.3–1.9)	3.5 (3.1–3.9)	22.2 (21.3–23.2)
**Household income**								
<$25,000	50,076	3.9 (3.5–4.3)	6.1 (5.6–6.6)	14.7 (13.9–15.6)	9.1 (8.5–9.6)	1.9 (1.7–2.2)	5.0 (4.5–5.5)	26.7 (25.7–27.7)
$25,000–$49,999	75,651	3.3 (3.0–3.6)	3.3 (3.0–3.5)	8.9 (8.4–9.4)	5.3 (5.0–5.6)	1.1 (1.0–1.3)	2.6 (2.4–2.9)	18.0 (17.4–18.6)
$50,000–$74,999	62,037	2.9 (2.6–3.2)	2.1 (1.8–2.4)	5.6 (5.2–6.1)	4.2 (3.8–4.7)	0.8 (0.6–1.0)	1.7 (1.5–2.0)	13.4 (12.7–14.1)
≥$75,000	164,319	2.2 (2.1–2.4)	1.1 (1.0–1.2)	3.4 (3.2–3.6)	2.6 (2.4–2.8)	0.5 (0.4–0.6)	1.0 (0.9–1.1)	8.7 (8.4–9.0)
Unknown	43,058	3.4 (3.0–3.9)	3.5 (3.1–4.0)	8.7 (8.1–9.4)	5.2 (4.7–5.7)	1.1 (0.9–1.4)	3.1 (2.7–3.6)	17.9 (16.9–18.9)

* Respondents were asked, “Are you deaf or do you have serious difficulty hearing?” (hearing); “Are you blind or do you have serious difficulty seeing, even when wearing glasses?” (vision); “Because of a physical, mental, or emotional condition, do you have serious difficulty concentrating, remembering, or making decisions?” (cognitive); “Do you have serious difficulty walking or climbing stairs?” (mobility); “Do you have difficulty dressing or bathing?” (self-care); and “Because of a physical, mental, or emotional condition, do you have difficulty doing errands alone such as visiting a doctor's office or shopping?” (independent living). Respondents who refused to answer, reported “don't know,” and had other missing responses were excluded from the analyses.

^†^ Respondents reported being either “employed for wages” or “self-employed” at the time of the interview, excluding active duty military or those missing information for occupation.

^§^ Unweighted.

^¶ ^Each disability type might not be independent; one respondent might have two or more disability types.

Prevalence of disability was highest in food preparation and serving-related (24.7%) and personal care and service (22.8%) occupation groups and lowest in the architecture and engineering group (8%) (Table 2). After adjustment for demographic characteristics (Table 3), occupation groups with the highest disability prevalences were food preparation and serving-related (19.9%); personal care and service (19.4%); and arts, design, entertainment, sports, and media (17.7%). Disability prevalences were lowest for business and financial operations (11.3%), health care practitioners and technicians (11.1%), and architecture and engineering (11.0%) (Table 3). The highest prevalences of specific disability types were in food preparation and serving-related for vision (4.2%), personal care and service for mobility (6.0%), and arts, design, entertainment, sports, and media for cognitive (10.8%). The prevalence of hearing disability was highest for the following occupational groups: installation, maintenance, and repair (4.2%); construction and extraction (3.8%); production (3.5%); protective services (3.5%); and farming, fishing, and forestry (3.5%).

**TABLE 2:** Unadjusted, weighted prevalence estimates of any disability and disability type* among currently employed^†^ U.S. adults aged 18–64 years, by major occupation groups^§^ — Behavioral Risk Factor Surveillance System, 35 states and Guam, 2016–2020

Major occupation group	No. of respondents^¶^	Disability type**														
		Hearing		Vision		Cognitive		Mobility		Self-care		Independent living		Any	
		Rank^††^	% (95% CI)	Rank^††^	% (95% CI)	Rank^††^	% (95% CI)	Rank^††^	% (95% CI)	Rank^††^	% (95% CI)	Rank^††^	% (95% CI)	Rank^††^	% (95% CI)
Management	46,710	10	2.7 (2.3–3.1)	18	1.3 (1.1–1.6)	18	3.9 (3.5–4.4)	14	3.6 (3.2–4.0)	14	0.7 (0.5–1.0)	16	1.4 (1.1–1.7)	15	10.3 (9.7–11.0)
Business and financial operations	17,691	21	1.6 (1.2–2.1)	20	1.2 (0.9–1.6)	17	4.1 (3.5–4.7)	17	2.9 (2.5–3.5)	18	0.6 (0.4–0.9)	15	1.6 (1.2–2.0)	19	9.3 (8.4–10.2)
Computer and mathematical	12,290	18	1.7 (1.3–2.3)	21	1.1 (0.8–1.6)	18	3.9 (3.3–4.6)	21	2.4 (1.8–3.1)	20	0.5 (0.3–0.7)	20	1.0 (0.8–1.3)	21	8.7 (7.7–9.8)
Architecture and engineering	11,076	13	2.2 (1.7–2.7)	22	0.9 (0.7–1.3)	22	2.9 (2.2–3.8)	17	2.9 (2.0–4.2)	14	—^§§^	20	1.0 (0.6–1.7)^¶¶^	22	8.0 (6.8–9.4)
Life, physical, and social science	5,913	14	2.1 (1.2–3.6)^¶¶^	16	1.6 (0.9–2.8)^¶¶^	15	4.7 (3.5–6.4)	22	1.6 (1.0–2.5)^¶¶^	11	0.8 (0.4–1.4)^¶¶^	17	1.2 (0.7–2.1)^¶¶^	17	9.7 (7.9–12.0)
Community and social services	9,220	18	1.7 (1.2–2.4)	13	1.9 (1.4–2.8)	13	5.5 (4.6–6.5)	11	4.8 (3.9–6.0)	11	0.8 (0.5–1.2)^¶¶^	14	1.8 (1.3–2.4)	13	12.4 (11.0–14.0)
Legal	4,768	21	1.6 (1.1–2.5)^¶¶^	14	1.7 (1.1–2.5)^¶¶^	21	3.5 (2.5–4.9)	20	2.8 (1.9–4.2)	22	0.3 (0.2–0.5)^¶¶^	22	0.8 (0.5–1.3)^¶¶^	20	9.2 (7.5–11.1)
Education, training, and library	29,714	18	1.7 (1.4–2.1)	18	1.3 (1.1–1.5)	16	4.6 (4.1–5.2)	15	3.4 (2.9–3.8)	18	0.6 (0.4–0.8)	17	1.2 (0.9–1.5)	16	9.9 (9.1–10.8)
Arts, design, entertainment, sports, and media	7,503	16	1.8 (1.4–2.4)	10	2.8 (2.0–4.0)	6	9.2 (7.7–10.9)	15	3.4 (2.8–4.3)	14	0.7 (0.5–1.1)^§§^	6	2.7 (2.1–3.4)	11	15.3 (13.5–17.4)
Health care practitioners and technicians	33,670	16	1.8 (1.4–2.2)	16	1.6 (1.3–1.9)	20	3.8 (3.2–4.4)	17	2.9 (2.6–3.3)	20	0.5 (0.4–0.6)	19	1.1 (0.9–1.4)	18	9.5 (8.6–10.5)
Health care support	9,627	14	2.1 (1.7–2.7)	5	4.3 (3.5–5.2)	4	10.9 (9.5−12.4)	3	6.9 (6.0–8.0)	9	1.1 (0.8–1.5)	3	3.6 (2.9–4.5)	4	20.0 (18.2–21.8)
Protective service	8,509	6	3.7 (2.7–4.9)	14	1.7 (1.2–2.4)	14	5.3 (4.2–6.7)	13	4.3 (3.4–5.4)	14	0.7 (0.4–1.2)^¶¶^	13	1.9 (1.3–2.7)	13	12.4 (10.7–14.3)
Food preparation and serving related	13,574	8	3.1 (2.6–3.7)	1	5.8 (4.8–7.1)	1	13.7 (12.5–15.1)	4	6.1 (5.4–6.9)	3	1.5 (1.2–2.0)	1	4.4 (3.7–5.3)	1	24.7 (23.1–26.4)
Building and grounds cleaning and maintenance	15,296	5	3.8 (3.2–4.7)	3	5.2 (4.5–6.1)	3	11.1 (10.1–12.3)	1	8.3 (7.4–9.2)	2	1.6 (1.3–2.1)	4	3.5 (2.9–4.2)	3	22.7 (21.2–24.3)
Personal care and service	12,249	9	2.8 (2.2–3.7)	4	4.8 (4.0–5.8)	2	11.7 (10.5–13.1)	2	7.6 (6.5–9.0)	4	1.4 (1.0–1.8)	2	4.3 (3.4–5.4)	2	22.8 (20.9–24.8)
Sales and related	35,095	11	2.5 (2.1–2.9)	12	2.6 (2.2–3.0)	5	9.6 (8.9–10.4)	12	4.5 (4.1–4.9)	10	0.9 (0.7–1.2)	5	2.9 (2.5–3.4)	9	16.5 (15.5–17.5)
Office and administrative support	37,977	12	2.4 (2.0–2.8)	11	2.7 (2.3–3.1)	12	7.2 (6.6–7.8)	6	5.2 (4.7–5.7)	11	0.8 (0.7–1.0)	8	2.6 (2.2–3.1)	12	14.9 (14.0–15.8)
Farming, fishing, and forestry	4,046	4	4.3 (3.1–6.0)	2	^—§§^	11	7.3 (5.4–9.8)	5	5.5 (3.4–8.8)^¶¶^	1	^—§§^	8	2.6 (1.4–4.6)^¶¶^	5	19.7 (15.8–24.4)
Construction and extraction	26,217	2	5.0 (4.4–5.7)	6	3.5 (2.9–4.2)	9	7.6 (6.9–8.4)	9	5.1 (4.5–5.8)	7	1.2 (1.0–1.5)	11	2.2 (1.9–2.6)	7	17.8 (16.7–18.9)
Installation, maintenance, and repair	15,034	1	5.9 (5.0–6.8)	7	3.1 (2.6–3.8)	10	7.4 (6.4–8.4)	10	5.0 (4.4–5.8)	4	1.4 (1.1–1.9)	6	2.7 (1.9–3.8)	6	18.0 (16.6–19.4)
Production	18,406	3	4.4 (3.9–5.0)	7	3.1 (2.6–3.7)	7	7.9 (7.1–8.9)	6	5.2 (4.6–5.9)	7	1.2 (0.9–1.6)	10	2.5 (2.1–2.9)	8	17.2 (16.1–18.4)
Transportation and material moving	20,556	6	3.7 (3.2–4.3)	9	3.0 (2.6–3.5)	8	7.8 (7.0–8.6)	6	5.2 (4.6–5.9)	6	1.3 (1.0–1.8)	12	2.0 (1.6–2.4)	9	16.5 (15.5–17.6)

**Abbreviation:** SOC = Standard Occupational Classification.

* Respondents were asked, “Are you deaf, or do you have serious difficulty hearing?” (hearing); “Are you blind, or do you have serious difficulty seeing, even when wearing glasses?” (vision); “Because of a physical, mental, or emotional condition, do you have serious difficulty concentrating, remembering, or making decisions?” (cognitive); “Do you have serious difficulty walking or climbing stairs?” (mobility); “Do you have difficulty dressing or bathing?” (self-care); and “Because of a physical, mental, or emotional condition, do you have difficulty doing errands alone such as visiting a doctor's office or shopping?” (independent living). Respondents who refused to answer reported “don't know,” and had other missing responses were excluded from the analyses.

^†^ Respondents reported being either “employed for wages” or “self-employed” at the time of the interview, excluding active duty military or those missing information for occupation.

^§ ^Twenty-two two-digit SOC major occupation groups (excluding military specific occupation group).

^¶ ^Unweighted.

** Each disability type might not be independent; one respondent might have two or more disability types.

^††^ Occupation groups ranked in order of descending prevalence of disability type or any disability (highest prevalence = 1 to lowest prevalence = 22); rankings not indicative of statistical significance.

^§§^ Estimates suppressed because the relative SE is >30%.

^¶¶^ Estimates might be unstable because the relative SE is 20%–30%.

**TABLE 3:** Adjusted,* weighted prevalence estimates of any disability and disability type^†^ among currently employed^§^ adults aged 18–64 years, by major occupation groups^¶^ — Behavioral Risk Factor Surveillance System, 35 states and Guam, 2016–2020

Major occupation group	No. of respondents**	Disability type^††^													
		Hearing		Vision		Cognitive		Mobility		Self-care		Independent living		Any	
		Rank^§§^	% (95% CI)	Rank^§§^	% (95% CI)	Rank^§§^	% (95% CI)	Rank^§§^	% (95% CI)	Rank^§§^	% (95% CI)	Rank^§§^	% (95% CI)	Rank^§§^	% (95% CI)
Management	46,710	14	2.5 (2.2–2.9)	19	1.7 (1.4–2.0)	20	5.0 (4.4–5.5)	17	3.8 (3.4–4.3)	15	0.8 (0.6–1.1)	18	1.7 (1.4–2.1)	18	11.8 (11.1–12.6)
Business and financial operation	17,691	21	1.9 (1.4–2.6)	21	1.6 (1.2–2.1)	17	5.3 (4.5–6.2)	20	3.3 (2.8–3.9)	15	0.8 (0.5–1.2)^¶¶^	13	1.9 (1.5–2.5)	20	11.3 (10.2–12.5)
Computer and mathematical	12,290	22	1.8 (1.3–2.5)	19	1.7 (1.3–2.4)	15	5.8 (4.9–6.9)	18	3.6 (2.8–4.6)	20	0.7 (0.5–1.0)	19	1.6 (1.3–2.1)	18	11.8 (10.4–13.3)
Architecture and engineering	11,076	18	2.1 (1.7–2.7)	22	1.5 (1.1–2.1)	22	4.4 (3.3–5.9)	11	4.4 (3.1–6.2)	9	—***	15	1.8 (1.0–3.0)^¶¶^	22	11.0 (9.3–13.0)
Life, physical, and social Science	5,913	11	2.6 (1.4–4.6)^¶¶^	9	2.7 (1.5–4.7)^¶¶^	9	7.0 (5.2–9.6)	22	2.3 (1.4–3.7)^¶¶^	3	1.2 (0.7–2.2)^¶¶^	15	1.8 (1.0–3.2)^¶¶^	13	13.7 (11.1–17.0)
Community and social services	9,220	15	2.4 (1.7–3.4)	10	2.6 (1.8–3.7)	7	7.4 (6.2–8.7)	2	5.9 (4.8–7.4)	6	1.1 (0.6–1.8)^¶¶^	9	2.3 (1.7–3.1)	7	16.1 (14.2–18.1)
Legal	4,768	18	2.1 (1.3–3.1)^¶¶^	10	2.6 (1.7–3.9)^¶¶^	18	5.2 (3.7–7.4)	19	3.5 (2.3–5.1)^¶¶^	22	—***	22	1.1 (0.7–1.8)^¶¶^	17	12.3 (10.0–15.1)
Education, training, and library	29,714	15	2.4 (2.0–3.0)	18	1.8 (1.5–2.1)	14	6.3 (5.6–7.1)	14	4.2 (3.6–4.7)	15	0.8 (0.6–1.2)	20	1.5 (1.2–1.9)	16	12.9 (11.9–14.1)
Arts, design, entertainment, sports, and media	7,503	18	2.1 (1.6–2.7)	3	3.6 (2.5–5.1)	1	10.8 (9.1−12.9)	15	4.1 (3.2–5.1)	13	0.9 (0.6–1.4)^¶¶^	1	3.1 (2.4–3.9)	3	17.7 (15.6–20.2)
Health care practitioners and technicians	33,670	17	2.3 (1.8–3.1)	16	2.0 (1.7–2.4)	21	4.5 (3.8–5.4)	21	3.2 (2.8–3.6)	21	0.6 (0.4–0.8)	21	1.2 (1.0–1.5)	21	11.1 (10.0–12.3)
Health care support	9,627	10	2.8 (2.2–3.5)	6	3.2 (2.6–4.0)	6	8.3 (7.3–9.5)	5	5.5 (4.8–6.3)	13	0.9 (0.6–1.3)	7	2.4 (1.9–3.0)	6	16.8 (15.2–18.4)
Protective service	8,509	3	3.5 (2.6–4.8)	17	1.9 (1.3–2.6)	16	5.7 (4.5–7.3)	7	5.0 (3.9–6.3)	15	0.8 (0.5–1.3)^¶¶^	11	2.2 (1.6–3.2)	15	13.2 (11.4–15.3)
Food preparation and serving related	13,574	6	3.4 (2.8–4.1)	1	4.2 (3.4–5.1)	2	9.2 (8.4−10.2)	3	5.8 (5.1–6.6)	1	1.3 (1.0–1.6)	4	2.8 (2.4–3.3)	1	19.9 (18.6–21.2)
Building and grounds cleaning and maintenance	15,296	8	3.3 (2.7–4.0)	5	3.3 (2.8–3.9)	4	8.6 (7.7–9.5)	4	5.6 (5.0–6.2)	6	1.1 (0.8–1.4)	5	2.7 (2.2–3.3)	4	17.3 (16.0–18.6)
Personal care and service	12,249	6	3.4 (2.5–4.5)	2	4.0 (3.2–4.9)	2	9.2 (8.2−10.3)	1	6.0 (5.0–7.1)	3	1.2 (0.9–1.6)	1	3.1 (2.4–4.0)	2	19.4 (17.8–21.2)
Sales and related	35,095	11	2.6 (2.2–3.1)	13	2.5 (2.2–2.9)	5	8.4 (7.8–9.1)	8	4.9 (4.5–5.3)	9	1.0 (0.8–1.3)	6	2.5 (2.1–2.9)	8	15.8 (14.9–16.8)
Office and administrative support	37,977	11	2.6 (2.2–3.2)	13	2.5 (2.2–2.9)	13	6.6 (6.0–7.2)	13	4.3 (3.9–4.7)	15	0.8 (0.6–1.0)	11	2.2 (1.8–2.7)	13	13.7 (12.9–14.6)
Farming, fishing, and forestry	4,046	3	3.5 (2.5–4.9)	4	3.4 (1.9–6.10)^¶¶^	18	5.2 (3.7–7.2)	15	4.1 (2.5–6.6)^¶¶^	3	—***	15	1.8 (1.0–3.2)^¶¶^	11	14.5 (11.9–17.7)
Construction and extraction	26,217	2	3.8 (3.3–4.3)	8	2.9 (2.3–3.5)	12	6.8 (6.1–7.5)	8	4.9 (4.3–5.6)	9	1.0 (0.8–1.2)	7	2.4 (2.0–2.9)	9	15.7 (14.7–16.8)
Installation, maintenance, and repair	15,034	1	4.2 (3.6–4.9)	7	3.0 (2.5–3.7)	8	7.3 (6.4–8.4)	6	5.3 (4.6–6.2)	1	1.3 (1.0–1.8)	1	3.1 (2.2–4.4)	5	17.0 (15.7–18.4)
Production	18,406	3	3.5 (3.0–3.9)	10	2.6 (2.2–3.0)	9	7.0 (6.3–7.9)	11	4.4 (3.9–5.0)	9	1.0 (0.7–1.3)	9	2.3 (1.9–2.7)	10	14.9 (13.9–16.0)
Transportation and material moving	20,556	9	2.9 (2.5–3.4)	15	2.4 (2.0–2.8)	9	7.0 (6.3–7.9)	10	4.6 (4.0–5.3)	6	1.1 (0.8–1.5)	13	1.9 (1.5–2.4)	11	14.5 (13.6–15.5)

**Abbreviation:** SOC = Standard Occupational Classification.

* Adjusted for age, sex, race and ethnicity, and education.

^† ^Respondents were asked, “Are you deaf or do you have serious difficulty hearing?” (hearing); “Are you blind or do you have serious difficulty seeing, even when wearing glasses?” (vision); “Because of a physical, mental, or emotional condition, do you have serious difficulty concentrating, remembering, or making decisions?” (cognitive); “Do you have serious difficulty walking or climbing stairs?” (mobility); “Do you have difficulty dressing or bathing?” (self-care); and “Because of a physical, mental, or emotional condition, do you have difficulty doing errands alone such as visiting a doctor's office or shopping?” (independent living). Respondents who refused to answer, reported “don't know,” and had other missing responses were excluded from the analyses.

^§ ^Respondents reported being either “employed for wages” or “self-employed” at the time of the interview, excluding active duty military or those missing information for occupation.

^¶^ Twenty-two two-digit SOC major occupation groups (excluding military specific occupation group).

** Each disability type might not be independent; one respondent might have two or more disability types.

^††^ Unweighted.

^§§^ Occupation groups ranked in order of descending prevalence of disability type or any disability (highest prevalence = 1 to lowest prevalence = 22); rankings not indicative of statistical significance.

^¶¶^ Estimates might be unstable because the relative SE is 20%–30%.

*** Estimates suppressed because the relative SE is >30%.

## Discussion

This report is the first to examine differences in disability prevalence by occupation group and includes adjustment for age, sex, race and ethnicity, and education. Persons with disability face employment disparities, a multidimensional issue involving barriers to finding and keeping jobs, including non-inclusive recruitment and hiring practices, lack of workplace communication and support, discrimination, and reduced workplace opportunities (*4**,**5*). Although the Americans with Disabilities Act protects the rights of most persons with disability who are employed or seeking jobs, additional resources could do more to shift attitudes and improve workplace equity (*6**)*. The higher percentage of persons with disability in service occupations (e.g., personal care and food preparation) might reflect better workplace programs, employees self-selecting into these jobs on the basis of perceived skill levels, less competition for these generally lower-wage jobs ($29,450–$33,620 mean annual wage compared with $58,260 for all occupations***), and other factors. Understanding differences in disability prevalence within and among occupation groups can help focus interventions and future research.

The proportion of working adults who reported a disability was highest among young adult workers and declined by age. This finding primarily reflects higher prevalences of cognitive disability among these younger workers. The ascertainment or prevalence of cognitive disabilities, which include autism spectrum differences, attention-deficit/hyperactivity disorder, and intellectual disability, has increased in recent years, particularly among children and adolescents; this finding might explain the higher prevalence of self-reported cognitive disabilities among young adult workers in the current study (*7*). With early diagnosis and interventions, young persons with disability are likely better positioned for productive employment and successful integration into the workforce (*8*). Alternatively, the declining prevalence of cognitive disability in older age groups might reflect longer continued employment among workers without congenital or acquired cognitive disabilities.

The highest prevalences of hearing disability were reported among five occupation groups: installation, maintenance, and repair; construction and extraction; production; farming, fishing, and forestry; and protective service. Several occupations within these groups involve loud work processes and equipment that increase the risk for occupational noise exposure (*9*); findings in these groups might be linked to the higher rates of occupational hearing loss. During 2006–2010, prevalence and incidence of occupational hearing loss was highest for mining, which encompasses many extraction occupations and construction industries (*9*). In addition, limited hearing function might not be a substantial entry barrier for these occupations. Hearing conservation programs and use of hearing protection might be important for these occupation groups.

The findings in this report are subject to at least five limitations. First, BRFSS data are self-reported, and recall or social desirability bias might influence the responses. Second, BRFSS data are cross-sectional, so temporality and causality cannot be inferred, and the work-relatedness of any reported disability is unknown. Third, the major occupation groups are broad and include component occupations with differing disability profiles. Fourth, the data do not allow differentiation of part-time and full-time workers. Finally, the results are not nationally representative; data were available from 35 states and Guam.

Employer measures to increase workplace accessibility measures and training designed to meet the needs of employees with disability might broaden the range of occupations in which these workers can succeed. To support employment of persons with disability, the U.S. Department of Labor sponsors technical assistance resources including the Employer Assistance and Resource Network on Disability Inclusion and Partnership on Employment and Accessible Technology.^†††^ These programs offer services to help employers integrate persons with disability into the workforce, including a framework with strategies for building a disability-inclusive organization and improving workplace access to new and emerging technologies. An increase in home-based, part-time, and computer-based jobs during the previous decade has provided a wider variety of job types for persons with disability (*3*). Improving access to computer-based technologies for persons with disability could further this progress and increase the availability of higher-wage, skilled jobs. According to the Job Accommodation Network, approximately one half of job accommodations cost employers nothing, and three fourths of implemented job accommodations were found to be very or extremely effective.^§§§^ Additional research is needed to improve understanding of how employers can improve disability practices, including accommodations, interventions, and programs to promote the hiring and retention of employees with disability. Both employees with disabilities and employers can benefit from a more equitable and inclusive workforce.

**Summary**

**What is already known about this topic?**

Persons with disability are less likely to be employed compared with persons without disability due to various barriers related to hiring practices, training opportunities, and daily working experiences.

**What is added by this report?**

During 2016–2020, 14.8% of currently employed U.S. adults aged 18–64 years from 35 states and Guam reported having a disability. Among 22 major occupation groups, the prevalence of any disability was highest for food preparation and serving-related professions (19.9%).

**What are the implications for public health practice?**

Workplace programs that address the training, education, and workplace needs of employees with disability might improve workers’ ability to enter, thrive in, and advance in a wider range of occupations.

## Footnotes

*
https://www.cdc.gov/brfss/about/index.htm

† States and territories contributing data for at least 1 year during 2016–2020: Alaska, California, Colorado, Connecticut, Delaware, Florida, Georgia, Hawaii, Idaho, Illinois, Iowa, Kansas, Louisiana, Maryland, Massachusetts, Michigan, Minnesota, Mississippi, Missouri, Montana, Nebraska, New Hampshire, New Jersey, New Mexico, New York, North Carolina, North Dakota, Pennsylvania, Rhode Island, South Carolina, Tennessee, Texas, Vermont, Washington, Wisconsin, and Guam.

§ https://www.cdc.gov/brfss/annual_data/2016/pdf/2016-sdqr.pdf; https://www.cdc.gov/brfss/annual_data/2017/pdf/2017-sdqr-508.pdf; https://www.cdc.gov/brfss/annual_data/2018/pdf/2018-sdqr-508.pdf; https://www.cdc.gov/brfss/annual_data/2019/pdf/2019-sdqr-508.pdf; https://www.cdc.gov/brfss/annual_data/2020/pdf/2020-sdqr-508.pdf

¶
https://www.cdc.gov/niosh/docs/2022-125/pdf/2022-125.pdf?id=10.26616/NIOSHPUB2022125

**
https://csams.cdc.gov/nioccs/HelpCodingSchemes.aspx

††
https://www.bls.gov/soc/2010/2010_major_groups.htm

§§
https://www.cdc.gov/brfss/data_documentation/pdf/BRFSS_Data_Users_Guide_on_Disability_Questions_2018-508.pdf

¶¶ 45 C.F.R. part 46.102(l)(2), 21 C.F.R. part 56; 42 U.S.C. Sect. 241(d); 5 U.S.C. Sect. 552a; 44 U.S.C. Sect. 3501 et seq.

***
https://www.bls.gov/oes/current/oes_nat.htm

†††
https://www.dol.gov/agencies/odep

§§§
https://askjan.org/topics/costs.cfm?cssearch
